# New aspects in the phase behaviour of poly-N-isopropyl acrylamide: systematic temperature dependent shrinking of PNiPAM assemblies well beyond the LCST

**DOI:** 10.1038/srep15520

**Published:** 2015-10-23

**Authors:** Irmgard Bischofberger, Veronique Trappe

**Affiliations:** 1 University of Fribourg, Department of Physics, CH-1700 Fribourg

## Abstract

We investigate the phase behaviour of aqueous dispersions of poly-N-isopropyl acrylamide (PNiPAM) microgels above their lower critical solution temperature (LCST) and find that beyond a well-defined concentration the systems exhibit a peculiar behaviour: the microgels assemble into space-spanning gels that shrink in time while maintaining the shape of the container in which they have been formed. Over a wide range of concentrations this shrinking behaviour is independent of PNiPAM concentration, but systematically depends on temperature in a temperature range significantly exceeding the LCST. The overall shrinking characteristics are consistent with those expected for scaffolds made of materials that exhibit thermal contraction. However, for the PNiPAM assemblies contraction is irreversible and can be as large as 90%. Such characteristics disclose complex interactions between fully collapsed PNiPAM and water well beyond the LCST, the origin of which has yet to be elucidated.

Poly-N-isopropyl acrylamide (PNiPAM) is a well-known thermosensitive polymer that dissolves in cold water but collapses to a globule or phase separates when the temperature is increased beyond the lower critical solution temperature (LCST) of *T*_*c*_ ≈ 32 °C^1^,^2^. This temperature is almost a material constant of PNiPAM in water; it is essentially independent of PNiPAM molecular weight or architecture and over a wide range of concentrations independent of PNiPAM concentration^3^,^4^,^5^,^6^. We recently argued that such independence was consistent with hydrophobic hydration being the main contribution governing the phase behaviour of PNiPAM at the LCST^7^. This agrees with the finding that water interacting by direct hydrogen bonds with PNiPAM is not entirely released at or beyond the LCST^8^,^9^,^10^. Indeed, the globular state of PNiPAM still contains a significant amount of water, the PNiPAM concentration within a globule reaching a limiting concentration of *c*_*a*_ ≈ 0.4 g/ml at *T*_*a*_ ~ 36 °C, which remains unchanged upon further increase in temperature^11^. Remarkably, the conjunction of reaching *c*_*a*_ at *T*_*a*_ appears to be a general feature of PNiPAM in water. Exploring the phase behaviour of PNiPAM at fairly low concentrations, *c* < 10^−4^ g/ml, a number of authors reported the existence of stable mesoglobules beyond *T*_*a*_^12^,^13^,^14^,^15^,^16^,^17^. These mesoglobules are spherical assemblies composed of several PNiPAM-chains that do not coarsen in time. As for the globular state of single chains the PNiPAM concentration within the mesoglobules is reported to be *c*_*a*_ ≈ 0.4 g/ml independent of temperature, as long as the temperature is maintained above *T*_*a*_^12^,^13^,^16^,^17^. This not only indicates that at *T*_*a*_ the concentration of *c*_*a*_ is reached irrespective of the actual state of the system, it also shows that this condition represents an arrest condition beyond which the PNiPAM-rich phase can no longer coarsen.

The origin of this arrest is not obvious. In general, the kinetic arrest of a phase separating polymer solution is due to an interference of the glass transition with the phase separation process^18^. Quenching a system to a temperature that induces phase separation leads to the formation of a polymer-rich and polymer-poor phase. If in the course of forming the polymer-rich phase the concentration of this phase reaches the critical conditions of a glass, the phase separation process is arrested, as the glassy arrest of the polymer-rich phase impedes further densification. However, for PNiPAM in water the glass transition temperature is reported to intersect the phase separation boundary at ~90 wt% PNiPAM, well above *c*_*a*_^19^,^20^. At *c*_*a*_ the glass transition temperature is about −50 °C, far below *T*_*a*_. Clearly, the classical scenario for arrested phase separation does not account for the arrest of phase separation in PNiPAM systems. Instead, the complex balance of water-PNiPAM interactions appears to set a limit for the maximal release of water from a PNiPAM-rich phase. Despite the fact that a PNiPAM system coarsens between *T*_*c*_ and *T*_*a*_, just like an ordinary polymer solution exhibiting phase separation, the density of the PNiPAM-rich phase never exceeds *c*_*a*_. Arrest is here determined by temperature rather than concentration.

Because coarsening is still possible between *T*_*c*_ and *T*_*a*_, much of the arrest phenomenology that has been reported can be explained by taking the temperature history of the sample into account. Indeed, the size of the mesoglobules formed in the low concentration range has been shown to depend on both the PNiPAM concentration and the temperature quench: the smaller the concentration and the larger the temperature quench the smaller the mesoglobules^12^,^13^,^15^,^16^,^17^,^21^. In reality, no temperature quench is instantaneous due to the finite time it takes for heat to transfer through a sample. The system therefore spends some time in the temperature interval between *T*_*c*_ and *T*_*a*_ when quenched from a temperature below *T*_*c*_ to one above *T*_*a*_. During this time domains of the PNiPAM-rich phase grow and merge with other domains. As this time is a decreasing function of the quench depth, which effectively sets the heat transfer rate, the final size of the mesoglobules obtained is smaller for deeper quenches, as observed experimentally^12^,^13^,^15^. Considering that the growth of domains depends on diffusion, this reasoning also accounts for the concentration dependent size of the mesoglobules.

At higher PNiPAM concentrations the phenomenology of arrested phase separation becomes more complex, ranging from fractal aggregates^22^ to a very peculiar type of gel that shrinks in time^23^,^24^,^25^. In this contribution we explore the parameters that govern this shrinking behaviour using PNiPAM microgels for our experiments.

## Experimental

Our PNiPAM microgels are synthesized following the procedure described by Senff and Richtering^6^ using a molar ratio of *N*-isopropyl acrylamide (NiPAM) to *N*,*N*’-methylenbisacrylamide (BIS) of 0.014. As the radical polymerisation of NiPAM also involves auto-crosslinking reactions^26^,^27^, this ratio should be considered as a lower limit of the crosslinking density. For purification the product is filtered through glass wool and extensively dialysed against water. Stock solutions are then prepared by centrifugation, where we determine the concentration of the stock solutions by drying a given amount of the stock to obtain the PNiPAM microgel content as residual weight.

**Figure 1:**
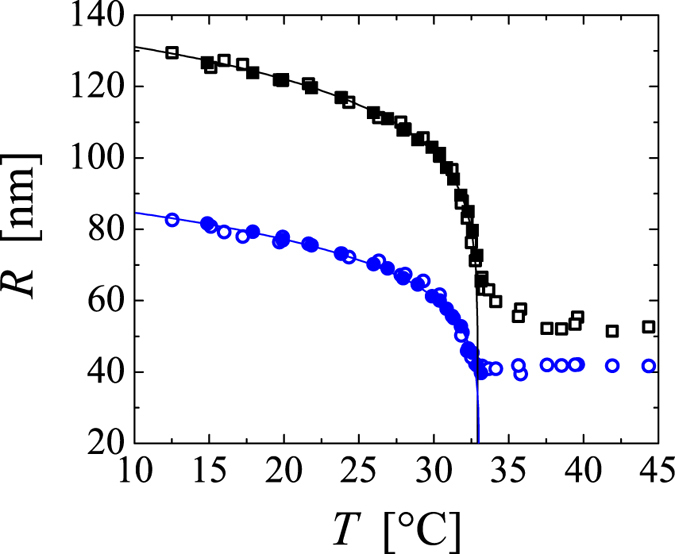
Temperature dependent dimensions of the PNiPAM microgels used in this work. Open symbols denote the radii obtained in pure water and filled symbols those obtained in 0.1 M NaSCN. The hydrodynamic radii, *R*_*h*_, are marked as black squares and the radii of gyration, *R*_*g*_, as blue circles. The error in *R*_*h*_ is ~4%, as determined from the scattering vector dependent variation of *R*_*h*_ in the range of scattering vectors *q* of 8.1∙10^6^ m^−1^ to 2.7∙10^7^ m^−1^. The error in *R*_*g*_ is ~5%, as determined from the standard deviation of the Guinier fits. The continuous lines through the low temperature data are fits to critical-like functions of the form *R*_*h,g*_ = *R*_*o*_ (1 − *T/T*_*c*_)^α^ yielding for *R*_*h*_: *T*_*c*_ = 33.0 °C, *R*_*o*_ = 137.2 nm, and *α* = 0.13; and for *R*_*g*_: *T*_*c*_ = 33.0 °C, *R*_*o*_ = 89.7 nm, and *α* = 0.16. Note that the dimensions of the fully collapsed state can only be determined at salt-free conditions, where due to electrostatic repulsions the microgels remain colloidally stable beyond *T*_*c*_.

Due to the use of an ionic initiator the microgels are charged. To screen these charges we add sodium thiocyanate (NaSCN) to our solutions, setting the salt concentration to 0.1 M. Our choice of NaSCN as screening agent is based on the sensitivity of PNiPAM to the Hofmeister effect, which generally leads to a decrease of the LCST when using kosmotropic salts such as NaCl^28^. This is not the case for chaotropic salts such as NaSCN; as denoted in Fig. 1, the temperature dependent dimensions of the PNiPAM microgels are independent of the NaSCN concentration. Note, however, that the dimensions of the fully collapsed state cannot be determined in the presence of salt, since the microgels aggregate above the LCST at these conditions. By contrast the collapsed microgels remain colloidally stable due to electrostatic repulsion at salt-free conditions. At these conditions we observe that the microgels further shrink beyond the LCST of *T*_*c*_ = 33 °C to reach their minimal dimension at *T*_*a*_ ~ 36 °C; at temperatures beyond *T*_*a*_ the size of the microgels remains constant.

For the characterization of the microgel dimensions we use a commercial goniometer set-up from ALV Langen to simultaneously perform static and dynamic light scattering experiments as a function of the scattering vector *q*. For these experiments we use a dilute microgel dispersion with a concentration of *c* = 4∙10^−5^ g/ml, which is prepared by diluting a stock solution of known concentration. From the time averaged scattering intensity *I*(*q*) the radius of gyration *R*_*g*_ is determined by using the Guinier approximation^29^ 
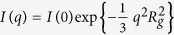
. From the intensity correlation functions determined in dynamic light scattering we determine the diffusion coefficient of the microgels, which we use to calculate the hydrodynamic radius *R*_*h*_ using the Stokes-Einstein equation. Consistent with work previously reported the two radii significantly differ from each other, which is due to an uneven distribution of crosslinks within the microgel^6^,^30^,^31^,^32^.

To fully explore the parameter space of arrested phase separation we prepare a series of samples by diluting a stock solution at a PNiPAM concentration of *c* = 3∙10^−2^ g/ml containing 0.1 M NaSCN with the appropriate amount of 0.1 M NaSCN solution. Note that our indication of concentration in g/ml refers to that obtained at 20 °C; for the concentration range investigated this approximately corresponds to the concentration in weight fraction. For the entire concentration series it is guaranteed that the microgels freely diffuse at 30 °C, which is the temperature at which we equilibrate the systems prior to a temperature quench beyond the LCST. Indeed, investigating the viscosity of the PNiPAM-systems as a function of concentration at 30 °C we find that the viscosity diverges at c* = 6∙10^−2^ g/ml, beyond the highest concentration of *c* = 5.3∙10^−2^ g/ml investigated (data not shown).

Our solutions are sealed in square sample cells (10 mm × 10 mm) and temperature quenches are performed by transferring the sample cell from a temperature bath set at 30 °C to a home-made temperature cell that is thermalized at the desired experimental set temperature with a precision of ±0.1 °C. For optical access, the temperature cell is equipped with two parallel glass windows. The arrested states of the system are primarily assessed by visual inspection two hours after the quench. However, to measure the temporal evolution of the size of the shrinking gels obtained at high concentrations we acquire images of the sample every 15 s after the temperature quench using a digital camera (Leica Digilux 2) over several hours.

Additional experiments to more accurately determine the phase boundary between stable mesoglobules and aggregated flocks are performed in dynamic light scattering. In the case of mesoglobule formation the intensity correlation functions remain almost perfectly exponential during and after the quench consistent with the formation of monodisperse assemblies. By contrast, in the case of the formation of flocks the intensity correlation functions become stretched, indicative of the formation of polydisperse assemblies.

## Results and Discussion

To place our work within the general context of PNiPAM aggregates, we investigate the phase behaviour of our microgels as a function of concentration and temperature. As described in the experimental section the quenches to *T* > *T*_*c*_ are performed by transferring the samples that are equilibrated in the dispersed state at 30 °C to a temperature cell thermalized at a set temperature beyond the LCST. The quench is thus not instantaneous but depends on the heat transfer across our 10 mm × 10 mm sample cells; the set temperature is typically reached after ~10 min.

**Figure 2:**
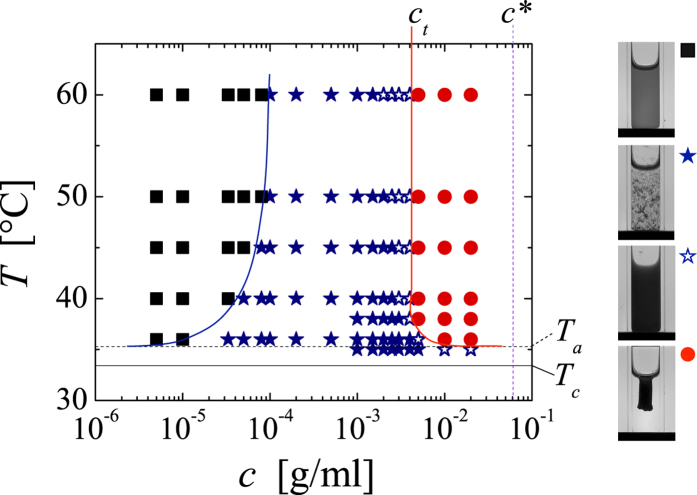
State diagram depicting the arrested states of PNiPAM microgels, as obtained two hours after a quench beyond the lower critical solution temperature *T*_*c*_. Phase separation of PNiPAM is always arrested beyond *T*_*a*_; with increasing concentration this leads to respectively stable mesoglobules (black squares), flocks (blue filled stars), space-filling aggregates (blue open stars) or shrinking gels (red circles). The blue line denotes the boundary between mesoglobules and flocculated states, as determined in dynamic light scattering experiments. The red line marks the onset to shrinking gels, as determined by visual inspection. The solid horizontal line indicates *T*_*c*_, the dashed horizontal line indicates *T*_*a*_. The dotted vertical line denotes the critical concentration c* beyond which the PNiPAM microgels are in a glassy state at 30 °C. The images on the right are representative examples for each state and are taken at *T* = 45 °C; from top to bottom: *c* = 1∙10^−5^ g/ml, 1∙10^−3^ g/ml, 3∙10^−3^ g/ml and 2∙10^−2^ g/ml. The errors in concentration are within 3%.

In the low concentration range, the system becomes turbid upon a quench, but remains homogeneously dispersed over extended periods of time, as shown in the top image of Fig. 2. At these conditions the microgels assemble into mesoglobules with characteristics similar to those observed for linear PNIPAM^12^,^13^,^15^,^16^,^17^,^21^: the deeper the quench (the higher the temperature) and the lower the concentration the smaller the size of the mesoglobule, as determined by dynamic light scattering (data not shown). At somewhat higher concentrations larger flocks form, as shown in the second image of Fig. 2. These flocks become space-filling assemblies withstanding gravitational collapse at larger concentrations, as shown in the third image of Fig. 2. All these states can be understood by considering that the PNiPAM microgels can at least partly fuse in the temperature interval between *T*_*c*_ and *T*_*a*_ to form mesoglobules, which in turn will aggregate to even larger objects, if the size of the mesoglobules exceeds a critical size^14^,^15^.

By contrast, beyond a well-defined concentration of *c*_*t*_ = 5∙10^−3^ g/ml the arrest of phase separation leads to the intriguing phenomenon of shrinking gels that is not evidently connected to the states observed at lower concentrations. For quenches to temperatures exceeding *T*_*a*_ the system evolves into a shrunken gel body that has the shape of the cell containing the sample; an example of such body is shown in the bottom image of Fig. 2. Because of interfacial effects the gel body is pinned at the air-water interface; when forced to fully immerse in the aqueous medium, this body sinks to the bottom of the container, which denotes that the density of the gel body is larger than that of the phase surrounding it. Testing the composition of this phase reveals that the phase ejected from the gel body is essentially pure water. Thus, shrinking does not involve a decrease of the PNiPAM content within the gel body.

**Figure 3:**
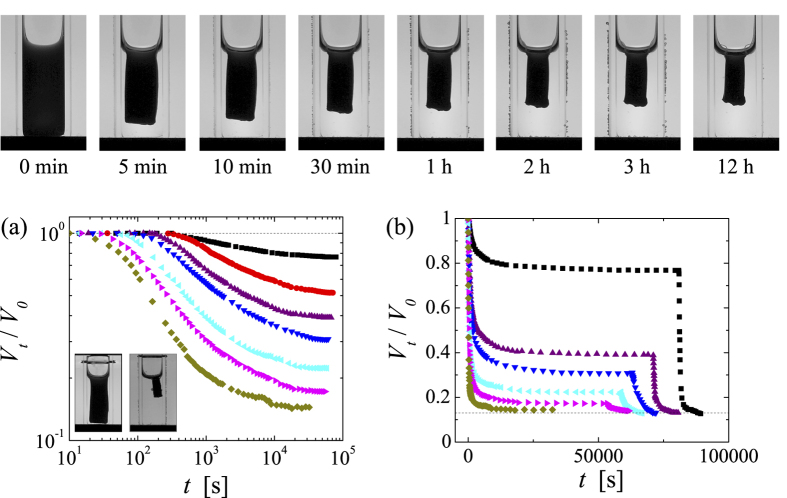
Temporal evolution of the gel dimensions. Top row: Images displaying the evolution of a system with *c* = 2∙10^−2^ g/ml after a quench to *T* = 45 °C. (**a**) Evolution of the gel volume *V*_*t*_ normalized by the initial sample volume *V*_*0*_ obtained for dispersions with *c* = 2∙10^−2^ g/ml that are quenched to: from top to bottom *T* = 36 °C, 37 °C, 38 °C, 40 °C, 45 °C, 50 °C, 60 °C. Inset: Images obtained at the end of the shrinking process for *T* = 36 °C (left) and *T* = 60 °C (right). (**b**) Response of gels with *c* = 2∙10^−2^ g/ml subjected to a two-step quench, *T*_*0*_ = 30 °C → *T*_*1*_ → *T*_*2*_ = 60 °C, with: from top to bottom *T*_*1*_ = 36 °C, 38 °C, 40 °C, 45 °C, 50 °C, 60 °C. The errors in *V*_*t*_/*V*_*0*_ are ±0.01.

The evolution of the system after a quench towards a shrunken gel body is shown in Fig. 3. As a typical example, we show the images obtained for a PNiPAM system with *c* = 2∙10^−2^ g/ml, which is submitted to a quench to *T* = 45 °C. Initially the gel fills all space and then gradually shrinks to a final size. As the gel retains the shape of the container throughout the shrinking process we can evaluate the volume occupied by the gel *V*_*t*_ from the gel area *A*_*t*_, which we determine digitally from the images acquired during the shrinking process. To compare different experiments we normalize *V*_*t*_ by the initial volume of the sample *V*_*0*_, *V*_*t*_/*V*_*0*_ = (*A*_*t*_/*A*_*0*_)^3/2^.

**Figure 4:**
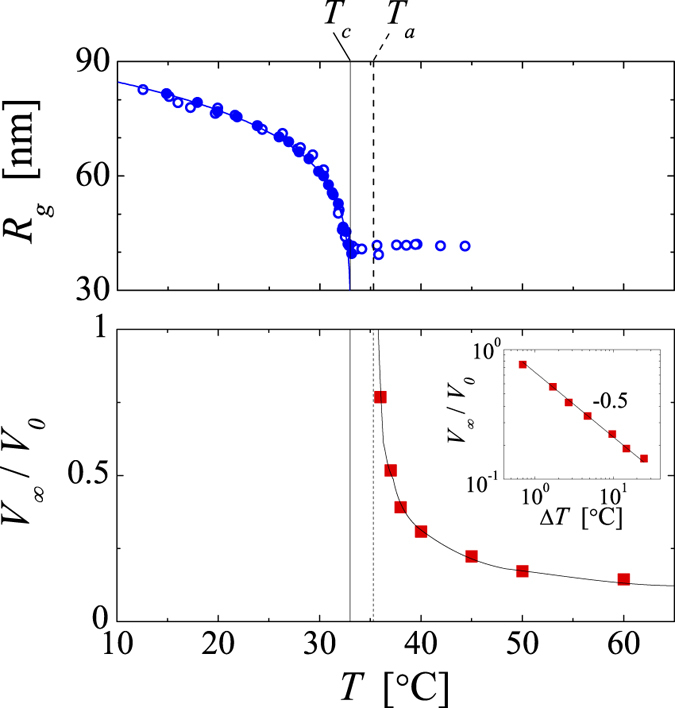
Shrinking of microgels and microgel assemblies occur at distinct temperature ranges. Top: Radius of gyration of PNiPAM microgels as a function of temperature, same data as shown in Fig. 1. Bottom: Normalized final gel volume *V*_∞_/*V*_*0*_ obtained from the long-time limit of the data shown in 3(a) as a function of temperature. The errors in *V*_∞_/*V*_*0*_ are ±0.01. The fit through the data is empirical: 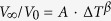 with Δ*T* = *T* – *T*_*a*_, *T*_*a*_ = 35.3 °C, *A* = 0.67 and *β* = −0.5. Inset: Double logarithmic plot of *V*_∞_/*V*_*0*_ as a function of the quench depth Δ*T*. The vertical solid and dotted lines indicate respectively *T*_*c*_ and *T*_*a*_.

As shown in Fig. 3(a), the shrinking process is a strong function of the quench temperature; the deeper the quench (the higher the temperature) the faster the shrinking process and the smaller the final size of the gel body. In particular for shallow quenches we observe that the onset to shrinking is somewhat delayed. However, accounting for the heating rate set by the transfer of our sample cells with an initial temperature of 30 °C to the temperature cell equilibrated at the set temperature, we find that this delay corresponds to the time it takes for the system to reach *T*_*a*_ ~ 36 °C. This is consistent with the fact that for quenches to set temperatures between the *T*_*c*_ and *T*_*a*_ shrinking is not observed. This, however, is rather peculiar. Indeed, the well-known internal shrinking upon increasing the temperature of any aqueous PNiPAM-system, shall it be a swollen gel, microgel or linear chain precisely stops at *T*_*a*_; beyond this temperature, no further densification is observed^3^,^6^,^11^. As shown in Fig. 1, this is also the case for the microgels under investigation. By contrast, for the assemblies composed of the already fully collapsed microgels shrinking sets in only if *T*_*a*_ is exceeded. Moreover, the amount of shrinking, the final size of the gel body *V*_*∞*_, changes considerably within the temperature range of 36–60 °C investigated, as shown in Fig. 4; within this temperature range none of the other aggregated states of PNiPAM exhibit any noticeable temperature dependence^12^,^16^. To emphasize these distinct temperature ranges we show the temperature dependence of radius of gyration of the PNiPAM microgels, already shown in Fig. 1, also in Fig. 4.

Most remarkably, the size of the gel body is not set by kinetics, as we may have naturally assumed. Increasing the temperature further after a given size has been reached leads to further shrinking, as shown in Fig. 3(b). The size of the gel body eventually reached corresponds to that obtained upon a direct quench. The final gel size seems thus uniquely set by the quench depth independent of the temperature history; for the range of temperatures investigated *V*_*∞*_ exhibits a power-law dependence with respect to Δ*T* = *T* − *T*_*a*_, as shown in the inset of Fig. 4, where the best fit to the data yields *T*_*a*_ = 35.3 °C and an exponent of −0.5. Further evidence that Δ*T* is the parameter governing the shrinking behaviour is given in the Supplementary Information, where we show the shrinking behaviour of a PNiPAM-system for which the arrest temperature has been shifted to lower temperatures by the addition of alcohol^7^,^33^; the shrinking behaviour of PNiPAM in the water/alcohol mixture is indistinguishable from that in water as long Δ*T* is maintained constant.

**Figure 5:**
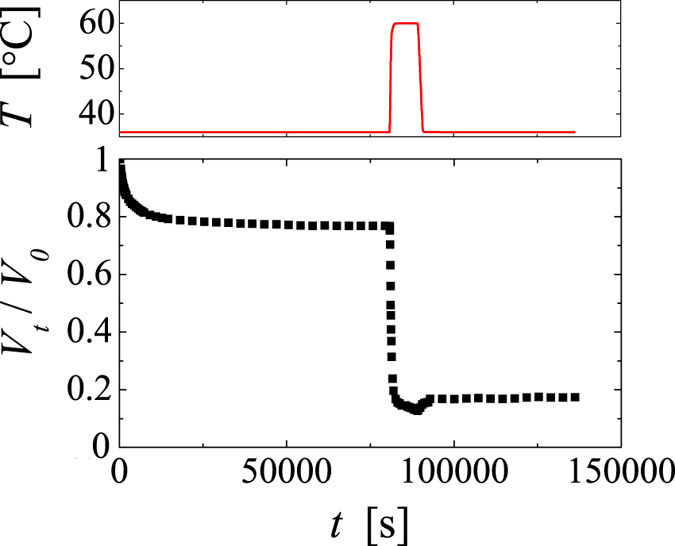
Response of a PNiPAM microgel solution with *c* = 2∙10^−2^ g/ml subjected to a temperature cycle from initially 30 °C to 36 °C → 60 °C → 36 °C. The temperature cycle is displayed in the upper panel. The errors in *V*_*t*_/*V*_*0*_ are ±0.01.

Such systematic dependence on Δ*T* is strangely reminiscent of equilibrium processes. However, the shrinking process is clearly non-equilibrium in nature, as it is not reversible unless the temperature is decreased below the LCST, whereupon the microgels redissolve to form a homogeneous dispersion. A given gel body obtained by an initial quench to a temperature *T* ≥ *T*_*a*_ will shrink further upon increasing the temperature further; however, it will not swell upon decreasing the temperature within the temperature range of *T* ≥ *T*_*a*_. This is shown in Fig. 5, where we report the response of a PNiPAM system with *c* = 2∙10^−2^ g/ml to a temperature cycle from initially 30 °C to 36 °C → 60 °C → 36 °C. Once the gel has shrunken to its final size at 60 °C, decreasing the temperature back to 36 °C leads only to a very small relaxation of the gel size, but clearly the gel does not relax back to the size it had after the first quench to 36 °C. Thus, the size of the gel is effectively set by the deepest quench ever experienced.

**Figure 6:**
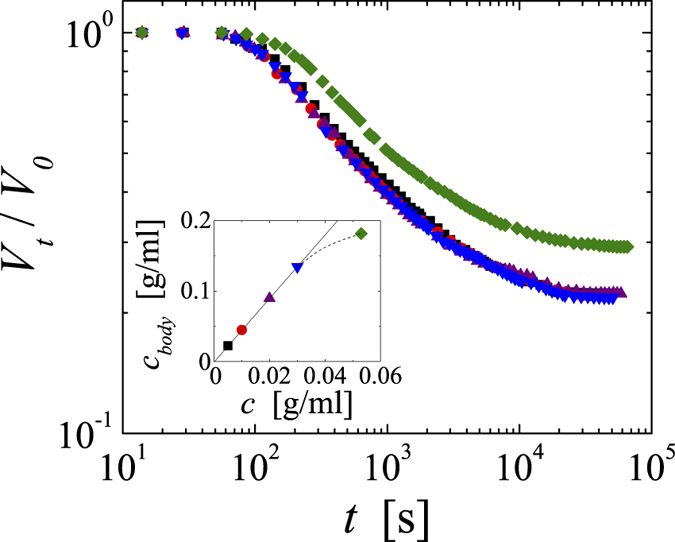
Evolution obtained upon a quench to *T* = 45 °C for systems with PNiPAM concentrations of: *c* = 5∙10^−3^ g/ml (squares), *c* = 10^−2^ g/ml (circles), *c* = 2∙10^−2^ g/ml (triangles up), *c* = 3∙10^−2^ g/ml (triangles down), *c* = 5.3∙10^−2^ g/ml (diamonds). Inset: PNiPAM concentration within the final shrunken gel body *c*_*body*_ = *c*/(*V*_*∞*_/*V*_*0*_) as a function of the PNiPAM concentration of the initial solution. The errors in *V*_*t*_/*V*_*0*_ are ±0.01.

Further adding to the puzzle of the shrinking process beyond *T*_*a*_, we find that over a rather wide range of concentration the temporal evolution and final size of the gel body is independent of PNiPAM concentration. As shown for quenches to *T* = 45 °C in Fig. 6, our PNiPAM-systems with concentrations varying by a factor of six exhibit the exact same shrinking behaviour. Deviations are only observed for systems with concentrations exceeding 3∙10^−2^ g/ml. Considering that all microgels are trapped within the gel body, this implies that the concentration of PNiPAM within the shrunken gel body, *c*_*body*_ = *c*/(*V*_*∞*_/*V*_*0*_), varies by a factor of six, as shown in the inset of Fig. 6.

**Figure 7:**
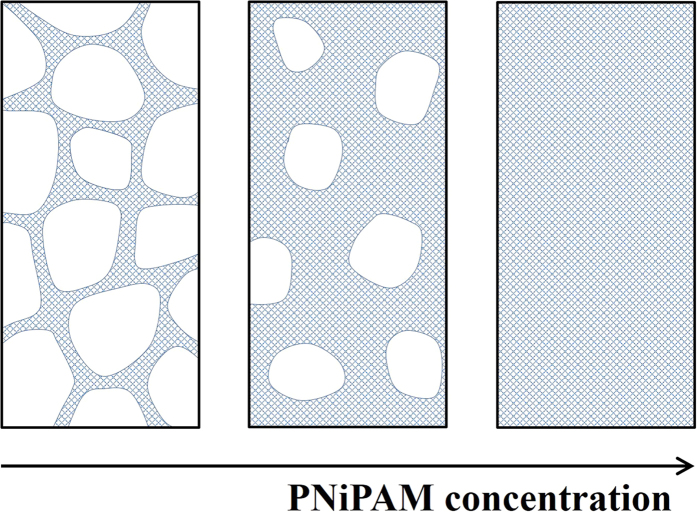
Scenario proposed to account for the concentration independent shrinking behaviour. The schematics illustrate the situation at *T*_*a*_ before shrinking sets in. The shrinking gels are scaffolds composed of a porous material denoted as grid pattern. This porous material has the same structural configuration at different concentrations and exhibits a temperature dependent contraction. The fraction of the volume occupied by the scaffold varies with PNiPAM concentration. The last panel represents the situation when the scaffold material fills all space: assuming that 90% of this material is made up of pores containing pure water the volume fraction occupied by the PNiPAM-rich phase is 0.1 and accounting for the fact that the PNiPAM concentration within the PNiPAM-rich phase is *c*_*a*_ = 0.4 g/ml the overall PNiPAM concentration is 0.04 g/ml. At lower PNiPAM concentration, the fraction of the volume occupied by the scaffold is lower than 1; the ‘empty’ space containing water is denoted in white. By locally contracting each volume element of the scaffold, the volume elements of the ‘empty’ space need to contract by the same amount. As a consequence, water from both the scaffold material and the ‘empty’ space is expelled from the gel body. Within this scenario the exact structural configuration of the scaffold is irrelevant; solely the concentration independent configuration of the scaffold material determines the shrinking. At large enough PNiPAM concentrations this material is forced to configure with a lower amount of water and the shrinking behaviour deviates form that obtained at lower concentrations where the scaffold material can freely configure at *T*_*a*_ (see Fig. 6).

We do not have a definite explanation for the observed shrinking phenomenon. However, our experimental findings permit for the discussion of the basic features of the shrinking. Indeed, this phenomenon bares striking similarities with thermal contraction of metals for instance. There the macroscopic shape of an object is maintained as well. More importantly, the degree of contraction of a metal body containing holes is independent of the actual volume fraction of holes; each volume element within the body contracts by the same amount, independent of whether the volume element is empty or filled with material. This is consistent with the shrinking behaviour of our PNiPAM gels, suggesting that they can be considered as simple scaffolds with varying volume fractions of ‘empty’ space accounting for the concentration independent behaviour, as sketched in Fig. 7. However, this implies that the structural rearrangements involved in the contraction of the scaffold elements must be very local, such that each volume element contracts individually. Based on the temperature-dependent behaviour of mesoglobules for instance we can ascertain that the PNiPAM-rich phase itself is not contracting beyond *T*_*a*_^12^,^15^,^16^,^17^. Thus, the scaffold element must contain both PNiPAM-rich domains, where the PNiPAM concentration is *c*_*a*_, and pure water. To denote such presumably microporous structure as scaffold material we use a grid pattern in our sketch of Fig. 7. From the degree of shrinking obtained at the deepest quench investigated, *T* = 60 °C, we can infer that the scaffold element must contain at least 85% of the pure water domains before shrinking sets in. Assuming a pure water content of 90% within a scaffold element before shrinking and using *c*_*a*_ = 0.4 g/ml to calculate the volume fraction of the PNiPAM-rich phase, we estimate that the critical concentration at which the scaffold elements would fill all space before shrinking is *c* ~ 0.04 g/ml. This is consistent with the limit of the concentration independent shrinking reported in Fig. 6 and indicates that once the scaffold elements can no longer contain a well-defined amount of water at the onset of shrinking the shrinking characteristics deviate from those observed when the scaffold element is free to configure with the appropriate amount of water.

The proposed scenario clearly entails that the contraction coefficient of the scaffold element is concentration independent; this in turn implies that the structure of this element is independent of concentration. Considering the behaviour observed at lower concentrations, this is difficult to understand. Indeed, below *c*_*t*_ the structural characteristics obtained upon arrest of phase separation are a strong function of the temperature history as well as concentration. None of this seems to apply to the shrinking gels. To fully test that the structures formed within the time interval between *T*_*c*_ and *T*_*a*_ do not impact the shrinking behaviour, we maintain a PNiPAM-system with *c* = 2∙10^−2^ g/ml at 34 °C for 24 hours allowing for significant coarsening before raising the temperature to 45 °C. Again we find that the shrinking behaviour is equivalent to that obtained by a direct quench. This suggests that the local structural configuration of the scaffold element that determines the contraction coefficient develops at *T*_*a*_ and that the driving force for the formation of this structure is also that determining the degree of shrinking at higher temperatures.

To fully account for the observed shrinking behaviour one certainly also needs to address the remarkable sharpness of the boundary delimiting the phase space of shrinking gels. This boundary is defined by a distinct concentration, *c*_*t*_ = 5∙10^−3^ g/ml, which is almost independent of temperature, as shown in Fig. 2. Note that *c*_*t*_ is more than an order of magnitude below the overlap concentration of the PNiPAM microgels at 30 °C, which is denoted as a vertical line in Fig. 2. Thus, the onset to shrinking gels is not due to a change in the initial condition of the system; for all quenches probed in this work it is guaranteed that the microgels freely diffuse prior to the quench. In reference to classical spinodal decomposition, one may envision that beyond *c*_*t*_ phase separation leads to the formation of interconnected networks from the very beginning of the phase separation process, where at *T*_*a*_ the actual structural configuration determining the shrinking behaviour is created within this network. We generally observe that the shrinking process of the shrinking gels involves the formation of bubbles, which may be indicative of a hindered water transport throughout the scaffold material.

Note that *T*_*a*_ marks both the limit of a water-expelling process on the molecular level and the onset of a water-expelling process on the mesoscopic level; the shrinking of a single microgel ceases at *T*_*a*_, while the shrinking of the assembled gels starts at *T*_*a*_. The origins of both shrinking processes are thus likely related. There is ample evidence that the LCST behaviour of PNiPAM is mainly governed by hydrophobic hydration^7^,^8^,^9^,^10^,^34^,^35^ and it is conceivable that the creation of the structural configuration of the scaffold element at *T*_*a*_ is due to hydrophobic effects as well. Within this context *T*_*a*_ would denote the transition between hydrophobic effects driving dewetting on molecular length-scales and those driving dewetting on mesoscopic length-scales.

Let us stress that our attempts to rationalize our experimental findings are purely speculative and remain to be proven. Fact is that the overall features of the shrinking behaviour indicate that the rearrangements involved must be very local, such that the overall shape of the gel body is maintained. In addition, the concentration independence of the shrinking behaviour as well as the independence on the temperature history of the sample denotes that the structural elements determining the contraction are not dependent on the structures formed during coarsening below *T*_*a*_. How exactly the structural elements, allowing for a contraction of as much as 90%, are configured and how and why they are formed are topics of future research.

## Conclusions

In this contribution we explored the phase behaviour of PNiPAM microgels beyond the lower critical solution temperature *T*_*c*_. Our investigations cover a wide range of concentrations, which enabled us to gain an overview of the characteristic states obtained at temperatures exceeding *T*_*c*_. In particular, we find that the phase space beyond *T*_*c*_ is characterized by two well-defined characteristic parameters: the arrest temperature *T*_*a*_ and the concentration *c*_*t*_ beyond which we observe the formation of gel bodies that shrink in time. Between *T*_*c*_ and *T*_*a*_ the system can evolve by phase separation and coarsen in time, while phase separation is arrested beyond *T*_*a*_. This applies to all concentrations. Above *T*_*a*_, however, the phase space is divided by *c*_*t*_. Below *c*_*t*_ the characteristics of the arrested states, mesoglobules and aggregates, are functions of the PNiPAM concentration and the temperature history of the sample or more precisely the time spent at the coarsening conditions between *T*_*c*_ and *T*_*a*_. By contrast, above *c*_*t*_ the phenomenology of shrinking gels, which are assemblies of fully collapsed microgels, is independent of these parameters. Instead, the degree of shrinking depends on the depth of the temperature quench defined as Δ*T* = *T* − *T*_*a*_, a parameter that is irrelevant for the arrested states formed below *c*_*t*_. The general features of the phase behaviour discussed here are not restricted to the PNiPAM microgel system studied in this work, but also apply to linear PNiPAM^1^,^12^,^13^,^15^,^16^,^22^,^24^. In particular the behaviour of shrinking gels is independent of whether we use PNiPAM microgels or linear PNiPAM, as shown in the Supplementary Information.

The overall characteristics of shrinking gels are reminiscent of those expected for scaffolds made of a material that thermally contracts, where the shape of the scaffold is maintained independently of the volume fraction occupied by the scaffold. However, for the shrinking gels the scaffold material allows for a contraction of 90%, which indicates that the relevant structural element is mainly composed of water. Since the shrinking behaviour is independent of concentration and of the degree of coarsening experienced within the temperature interval between *T*_*c*_ and *T*_*a*_ we conclude that the properties of the scaffold material are set at the arrest transition. The driving forces for the formation of this mainly water containing scaffold material are likely to be those determining the thermal contraction as well. The exact nature of these driving forces remains an open question.

## Additional Information

**How to cite this article**: Bischofberger, I. and Trappe, V. New aspects in the phase behaviour of poly-N-isopropyl acrylamide: systematic temperature dependent shrinking of PNiPAM assemblies well beyond the LCST. *Sci. Rep.* **5**, 15520; doi: 10.1038/srep15520 (2015).

## Supplementary Material

Supplementary Information
