# Supplementary material for: New aspects in the phase behaviour of poly-N-isopropyl acrylamide: systematic temperature dependent shrinking of PNiPAM assemblies well beyond the LCST
Source: Sci Rep. 2015 Oct 23;5:15520. doi: 10.1038/srep15520 (PMC4616040; doi:10.1038/srep15520)
Supplement: Supplementary Information [file srep15520-s1.pdf]

Supplementary Information

New aspects in the phase behaviour of poly-N-isopropyl acrylamide: systematic temperature dependent shrinking of PNiPAM assemblies well beyond the LCST

I. Bischofberger* and V. Trappe

University of Fribourg, Department of Physics, CH-1700 Fribourg

**current address: University of Chicago, Department of Physics, Chicago, IL 60637*

As inferred from the data shown in Figure 3(c) in the manuscript, the shrinking behaviour is set by $\Delta T = T - T_a$, where T is the quench temperature and T_a the temperature beyond which phase separation is arrested. Further evidence that ΔT is the parameter governing the shrinking behaviour is shown in Figure S1, where we compare the shrinking of two PNiPAM-systems with LCSTs and accordingly arrest temperatures that differ by 10°C. Maintaining ΔT fixed leads to identical shrinking behaviour.

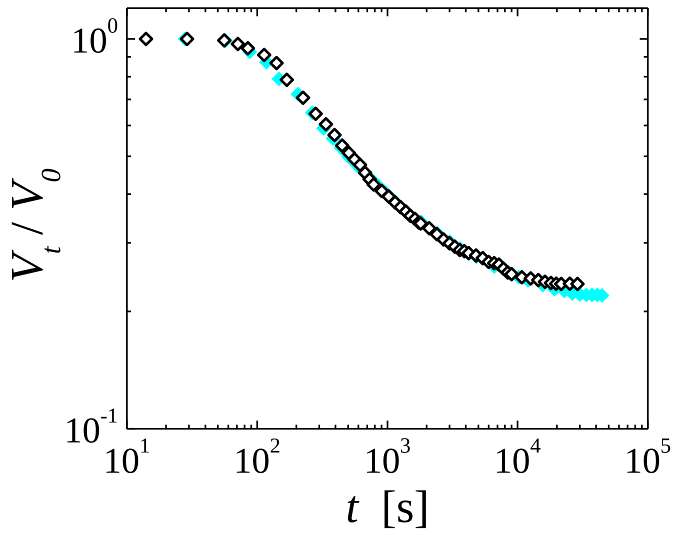

Figure S1: Shrinking behaviour obtained for a PNiPAM microgel solution in water subjected to a quench to $T = 45$°C (filled cyan symbols) compared to that obtained for a PNiPAM microgel solution in a water/ethanol mixture with an ethanol molar fraction of $X = 0.055$ subjected to a quench to $T = 35$°C (filled black symbols). In both solutions $c = 2{\cdot}10^{-2}$ g/ml and for both quenches $\Delta T \approx 9$°C. The errors in V_t/V_0 are ±0.01.

The shrinking of the gel body is not specific to the PNiPAM microgels discussed in the paper. As shown in Figure S2, a solution of linear PNiPAM chains exhibits the same shrinking behaviour as a solution of PNiPAM microgels. This shows that shrinking is a property of PNiPAM in general, independent of the PNiPAM architecture.

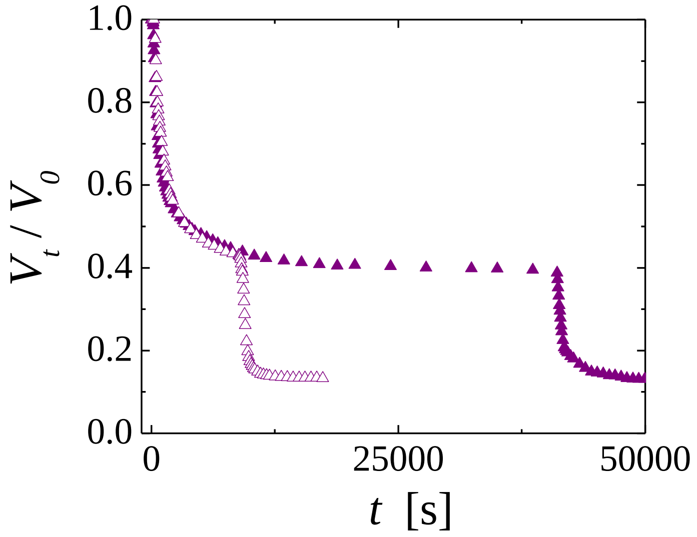

Figure S2: Shrinking behaviour obtained for aqueous solutions of PNiPAM microgels (filled symbols) and linear PNiPAM (open symbols). The linear PNiPAM has a viscosity averaged molecular weight of M_v = 465 500 g/mol and polydispersity of M_w/M_n = 4.9, as indicated by the supplier, Polymer Source Inc. Both systems equilibrated at 30°C are subjected to a two-step quench with first ΔT = 2°C and subsequently ΔT = 24°C. The second quench step is performed at t = 8800 s for the linear PNiPAM solution and at t = 41000 s for the PNiPAM microgel solution. The response of the gels to these quenches is identical for both PNiPAM systems, indicating that the formation of shrinking gels is a general feature of PNiPAM systems independent of the PNiPAM architecture.
